# Development of Novel SSR Markers for Flax (*Linum usitatissimum* L.) Using Reduced-Representation Genome Sequencing

**DOI:** 10.3389/fpls.2016.02018

**Published:** 2017-01-13

**Authors:** Jianzhong Wu, Qian Zhao, Guangwen Wu, Shuquan Zhang, Tingbo Jiang

**Affiliations:** ^1^State Key Laboratory of Tree Genetics and Breeding, Northeast Forestry UniversityHarbin, China; ^2^Institute of Industrial Crops, Heilongjiang Academy of Agricultural SciencesHarbin, China

**Keywords:** *Linum usitatissimum* L., genomic SSR, genetic diversity, flax, marker-assisted selection

## Abstract

Flax (*Linum usitatissimum* L.) is a major fiber and oil yielding crop grown in northeastern China. Identification of flax molecular markers is a key step toward improving flax yield and quality via marker-assisted breeding. Simple sequence repeat (SSR) markers, which are based on genomic structural variation, are considered the most valuable type of genetic marker for this purpose. In this study, we screened 1574 microsatellites from *Linum usitatissimum* L. obtained using reduced representation genome sequencing (RRGS) to systematically identify SSR markers. The resulting set of microsatellites consisted mainly of trinucleotide (56.10%) and dinucleotide (35.23%) repeats, with each motif consisting of 5–8 repeats. We then evaluated marker sensitivity and specificity based on samples of 48 flax isolates obtained from northeastern China. Using the new SSR panel, the results demonstrated that fiber flax and oilseed flax varieties clustered into two well separated groups. The novel SSR markers developed in this study show potential value for selection of varieties for use in flax breeding programs.

## Introduction

Flax (*Linum usitatissimum* L.) is a major fiber and oil crop grown in northeastern China. However, the lack of high quality fiber flax varieties adaptable to growth in this region is an unmet challenge of flax that exhibits higher yield and better quality fiber or oilseed is a top priority. Because marker-assisted selection (MAS) has helped to achieve similar goals efficiently for other crops, MAS should facilitate marker-assisted breeding of flax, as well as for flax germplasm identification. To date, several molecular markers has been identified in flax using isozyme analysis (Krulickova et al., 2002; Yurenkova et al., 2005), RAPD (Fu, 2006), AFLP (Spielmeyer et al., 1998; Everaert et al., 2001), and inter-simple sequence repeat (ISSR) analysis (Wiesner and Wiesnerová, 2003, 2004; Rajwade et al., 2010). However, development of markers using these methodologies has frequently been time-consuming, laborious and poorly reproducible.

More recently, a better approach utilizing simple sequence repeats (SSRs), or microsatellite DNA, has been developed. SSRs are short, tandemly repeating nucleotide motifs (1–6 bp long) that are widely distributed in genomes of eukaryotic organisms genomes including flax (Tautz, 1989; Temnykh et al., 2001). The abundance, highly polymorphic nature, heritability, distribution, reproducibility and generally co-dominant nature of SSR markers make them highly suitable for MAS and genetic diversity studies (Wiesner et al., 2001; Cloutier et al., 2011, 2012b; Soto-Cerda et al., 2011a; Kumar et al., 2015; Kessuwan et al., 2016).

In flax, large numbers of genomic SSR markers have already been developed (Cloutier et al., 2009, 2012a; Deng et al., 2010; Sandip et al., 2012). Three microsatellite isolation methods, all utilizing next-generation sequencing methodologies, have rapidly pinpointed SSR markers for evaluation of diverse oilseed flax genotypes. Such rapid advances demonstrate the value of this sequencing technology for SSR marker discovery (Sandip et al., 2012) and recent characterization among almost all fiber and oilseed flax cultivars (Roose-Amsaleg et al., 2006; Deng et al., 2010). Notably, 1506 putative simple sequence repeats have recently been assessed using a panel of 16 flax accessions, resulting in discovery of 818 novel polymorphic SSR primer pairs (Cloutier et al., 2012a). However, specific progress using these SSR makers toward the development of flax breeding programs must first await development of other necessary genetic and genomic resources.

Other recent advances in DNA sequencing have increased the availability of molecular markers, including genomic SSRs (Wiesner et al., 2001; Roose-Amsaleg et al., 2006; Deng et al., 2010; Soto-Cerda et al., 2011a; Cloutier et al., 2012a; Sandip et al., 2012) and SSRs from expressed sequence tags (EST-SSRs) (Cloutier et al., 2009, 2012a; Soto-Cerda et al., 2011b). Moreover, with the development of low-cost next generation sequencing technologies, RRGS has become a popular method for genotyping (Sun et al., 2013). RRGS, an alternative or complementary approach to complete genome sequencing, rapidly and inexpensively generates useful sequence data from large and complex genomes (Barbazuk et al., 2005).

Because high throughput technologies show great promise for SSR marker discovery, for RRGS, the Illumina sequencing platform was selected, due to its high throughput, relatively low cost, rapid results, and high accuracy (Kozich et al., 2013). We then applied RRGS to flax in order to rapidly and systematically identify SSR markers that would be immediately suitable for use in flax breeding. Using RRGS, only those genomic regions with repetitive sequences are selectively sequenced to identify new SSR markers. Next, 48 *Linum usitatissimum* cultivars/accessions were screened for SSR polymorphisms to assess the genetic diversity in these cultivars in preparation for marker-selected breeding.

## Materials and methods

### Plant materials

All plant materials for the 48 cultivars/accessions studied here, including the fiber cultivar “Diane,” were provided by the Germplasm Bank of the Institute of Industrial Crops of the Heilongjiang Academy of Agricultural sciences, China (Table 1).

**Table 1:** **List of flax cultivars used in the polymorphism analysis**.

**S. No**	**Cultivar**	**Source**	**Type**	**S. No**	**Cultivar**	**Source**	**Type**
1	Heiya14	China	Fiber	25	Shuangya1	China	Fiber
2	Shuangya7	China	Fiber	26	Shuangya14	China	Fiber
3	Huoju	Russia	Oilseed	27	Shuangya11	China	Fiber
4	AODILI-2	Austria	Oilseed	28	Shuangya3	China	Fiber
5	Y2012-291	China	Oilseed	29	MEROLIN	Netherlands	Fiber
6	Y2006-64	Britain	Oilseed	30	Heiya16	China	Fiber
7	CN00961	Canada	Oilseed	31	Heiya4	China	Fiber
8	Huaguang2	China	Oilseed	32	Agatha	France	Fiber
9	NEW1	Netherlands	Fiber	33	BO-1	Netherlands	Fiber
10	M0269-1	China	Oilseed	34	Jika	Chech	Fiber
11	CN40081	Canada	Oilseed	35	Diane	France	Fiber
12	M0298-4-6	China	Oilseed	36	Ariane	France	Fiber
13	SW-3	Sweden	Oilseed	37	Venus	France	Fiber
14	A0529	Russia	Oilseed	38	98001-6-3-11	China	Fiber
15	M03057-26	China	Oilseed	39	Hernus	France	Fiber
16	M0329-15-1	China	Oilseed	40	D93008-1-32	China	Fiber
17	Y2006-20	Russia	Oilseed	41	Adelie	France	Fiber
18	K-6	Russia	Fiber	42	Shuangya9	China	Fiber
19	K-1194	Russia	Fiber	43	Y2003-12	China	Oilseed
20	Y2003-43	China	Oilseed	44	Y2003-30	China	Oilseed
21	Y2012-307	China	Oilseed	45	Y2010-49	China	Oilseed
22	Y2012-304	China	Oilseed	46	Y0405-8-4	China	Oilseed
23	Shuangya8	China	Fiber	47	96021-6-62	China	Oilseed
24	Shuangya2	China	Fiber	48	Y2012-322	China	Oilseed

### DNA preparation

Samples of fresh, young leaf tissue at the first branching stage were collected and DNA was extracted using a One-tube Plant DNAup for PCR Kit (Sangon Biotech, Shanghai, China) according to the manufacturer's instructions. DNA quality and quantity were checked using 0.8% agarose gels and Eppendorf BioSpectrometer (Eppendorf, Hamburg, Germany), respectively. The DNA was further quantified using a fluorometer and diluted to a 10 ng/L working solution.

### Reduced representation genome sequencing (RRGs)

In the present study, we sequencing the fiber flax cultivar “Diane” genome using RRGS. The “Diane” cultivar, originally introduced from France, is a typical flax variety well adapted to growth in northeastern China. After construction of a genomic DNA library, shotgun sequencing was performed using the Illumina sequencing platform (HiSeq™ 2000) according to the manufacturer's instructions (Illumina, San Diego, CA) to generate 100 bp paired-end reads. After trimming to remove primer sequences, the short sequence reads were then assembled using SOAPdenovo software (Zhang et al., 2011). Since the flax genome assembly is currently limited to scaffolds, we mapped our short-sequence reads to published flax genome assembly scaffolds (http://www.ncbi.nlm.nih.gov/pubmed/22757964?dopt=Abstract).

### Identification of simple sequence repeats (SSRs)

The software MISA was used to identify SSRs with dimer, trimer, tetramer, pentamer, and hexamer motifs with lengths greater than 10 bp (Conradsen et al., 2009). In order to identify the novelty of SSRs developed in this study, all the sequences were aligned with highly similar sequences (megablast) in Nucleotide collection (Nt) database (https://blast.ncbi.nlm.nih.gov/Blast.cgi). And the sequences with matching degree above 80% were considered to be redundance.

### SSR primer pair design

The SSR primer pairs were designed using Primer Premier 5.0 software (Tu et al., 2011) using the following standard parameters: target amplicon length of 80–300 bp, annealing temperature variation from 55 to 65°C, GC content from 50 to 70%, and primer size of 18–28 bp. Three primer pairs were designed for each SSR locus, and the primer pair producing DNA with the highest score was chosen for further use in SSR marker studies. Primers were synthesized by GENEWIZ, Inc. (Suzhou, China). In order to test the effectiveness of the SSR set for classification of flax varieties, 62 loci out of the total collection of novel SSR markers were selected randomly as sites for subsequent genotypic testing of 48 cultivars ultimately for use in genetic diversity analysis (Table 1).

### PCR conditions for flax cultivar SSR polymorphism studies

Standard PCR was carried out in a reaction volume of 20 μl including 50 ng DNA, 1.0 μl of 10 μM forward primer, 1.0 μl of 10 μM reverse primer, 0.5 μl of 10 mM dNTP, 2 μl of 10X buffer (100 mM Tris–HCl, 500 mM KCl), 2 μl of 25 mM MgCl_2_, and 0.2 μl of Taq polymerase (5 U/μl). PCR amplification was performed using the following cycling conditions: Step 1, pre-cycling denaturation at 94°C for 2 min; Step 2, denaturation at 94°C for 30 s; Step 3, annealing at 55°C for 30 s; Step 4, extension at 72°C for 20 s. Steps 2–4 were repeated for another 34 cycles. The PCR products were separated on 8% non-denaturing polyacrylamide gel by electrophoresis at 280 V and 50W in 1X TBE buffer and visualized by 0.1% silver nitrate staining.

### Genetic diversity assay

After PCR, the presence or absence of bands in the gel images were visually scored “1” or “0” for each DNA sample after normalization of the original data using NTSYSpc2.11 software (Rohlf, 1997). Simple matching coefficients were calculated using the Qualitative Data Analysis Program (QDAP) (http://www.umass.edu/qdap/). Next, cluster analysis was conducted based on the unweighted pair-group method using an arithmetic averages (UPGMA) algorithm and the SAHN subroutine, both included in NTSYS-pc2.11 software using default options. The Tree plot module of the same software package was used to generate the dendrogram, and the color-coding bar and serial numbers attached to the dendrogram were drawn and modified manually.

The number of alleles and polymorphism information content (PIC) of the alleles revealed by each primer pair were calculated using Powermarker V3.25 (Liu and Muse, 2005) to generate the genotype data for the 48 accessions.

## Results

### Sequencing results and SSRS distribution in the flax genome

**Figure 1:**
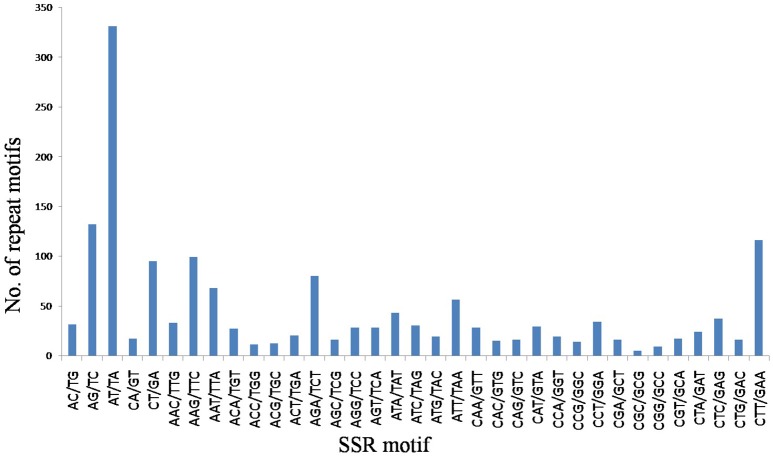
**Numbers of dinucleotide and trinucleotide SSRs classified based on their motifs**.

A total of 9.87 Mb reads were obtained with mapped reads reaching 78.14% and normal digestion ratio reaching 97.49%. SSR loci with 2–6 bp repeat motifs were identified from the sequencing data, and a total of 1720 SSR loci were identified (GenBank accession numbers: KY325484–KY327203) (Supplementary Table 1), which represented 20.53% of the total numbers of unigenes in the flax genome. Incidences of different repeat types and frequencies for each motif were evaluated based on the repeat unit number (Table 2). Among the SSR loci, 965 (56.10%) trinucleotide microsatellites demonstrated trinucleotide SSRs to be the most abundant microsatellite type, followed by 606 (35.23%) dinucleotide microsatellites. However, only 149 (8.67%) SSR loci contained other motifs, including 67 (3.9%) tetranucleotide repeats, 58 (3.37%) pentanucleotide repeats and 24 (1.40%) hexanucleotide repeats. The most common number of repeat iterations was five times (35.12%), followed by six times (28.08%), and seven times (14.53%). Of the six possible dinucleotide motifs, five motifs, namely AT/TA, AG/TC, CT/GA, AC/TG, and CA/GT, were represented, with AT/TA motifs most frequently represented, while CG/GC motifs were completely absent. Of the 30 possible trinucleotide motifs, CTT/GAA motifs were the most frequently represented trinucleotide microsatellites (Figure 1). Frequency distributions for tetranucleotide, pentanucleotide and hexanucleotide motif SSRs were more difficult to discern because they represent only a small proportion of the total SSRs detected.

**Table 2:** **Frequencies of different SSR repeat motif types**.

**SSR motif**	**Repeat number**								**Percentage (%)**
	**4**	**5**	**6**	**7**	**8**	**9**	**>9**	**Total**	
Dinucleotide			232	144	80	55	95	606	35.23
Trinucleotide		542	237	103	48	33	2	965	56.10
Quadnucleotide		52	12	3				67	3.90
Pentanucleotide	49	7	2					58	3.37
Hexanucleotide	21	3						24	1.40
Total	70	604	483	250	128	88	97	1720	

### Development and detection of genomic SSR markers

Statistical analysis showed that the identities of 146 (Supplementary Table 2) out of 1720 SSRs developed were higher than the threshold aligned with highly similar sequences in Nucleotide collection (Nt) database, therefore 1574 novel SSRs were developed ultimately in this study. Using the identified SSR loci, 62 primer pairs were designed (Supplementary Table 3) and their polymorphisms were identified by comparison of their sequences among 48 diverse cultivars (Table 1). A total of 1006 polymorphic DNA fragments were amplified from the 48 cultivars using 62 primer pairs (Supplementary Table 4). The polymorphism information content (PIC) was determined by both allele numbers and allele frequency distributions and was used to evaluate the variation of SSR alleles (Botstein et al., 1980). The results indicated that the 1006 loci had low to moderate PIC values, ranging from 0.06 to 0.5, with a mean of 0.39. The average number of alleles (Ne^*^) was 1.70 (Kimura and Crow, 1964), the average genetic diversity (Nei's gene diversity, H^*^) was 0.39 (Nei, 1973). The average information index (I^*^) was 0.57, and the frequency of the major genotypes (A^*^) varied from 0.20 to 0.97, with an average of 0.54.

## Discussion

In this study, we used RRGS technology to systematically identify 1574 specific genomic SSR loci, which were represented 91.5% of all the SSRs, and the others were redundant sequences to previous works. Moreover, abundant SSRs at single genetic loci were observed, which could be suitable for screening of flax specific markers, to facilitate development of a large number of flax SSRs at one time. Previously, 290 SSR markers had been identified from a large number of microsatellite motifs, 52 of which were used to evaluate linseed genotypes (Sandip et al., 2012). In this work, the large number of rapidly generated flax microsatellite markers demonstrates the efficiency of marker discovery using next-generation sequencing technology. Notably, this study resulted in validation of 62 randomly selected novel SSRs through phylogenetic clustering of fiber and oilseed flax cultivars. New polymorphic microsatellite loci are not only useful for screening cultivars from different sources (Cloutier et al., 2009; Deng et al., 2010), but also for identification of intraspecific relationship among *Linum* species (Soto-Cerda et al., 2011b). However, the comprehensive application of polymorphic markers to flax breeding programs has greatly lagged behind discoveries of new flax SSR markers. Polymorphic microsatellite loci are useful for genetic linkage map construction, germplasm classification and identification, gene identification and quantitative trait loci mapping, and marker-assisted in breeding of *L. usitatissimum*. However, SSR makers still have limited use in flax breeding programs, due to the lack of other genetic and genomic resources that must be developed before SSR markers can be utilized.

Previously, the development of flax SSR markers had mainly been based on expressed sequence tags (EST) (Cloutier et al., 2009, 2012a), but more recently, genomic SSR markers have been found to be the most polymorphic markers in flax (Cloutier et al., 2012b). In agreement with more recent results, this study demonstrated that the value of Nei's gene diversity per locus for genomic SSRs in flax was higher than that for EST–SSRs.

**Figure 2:**
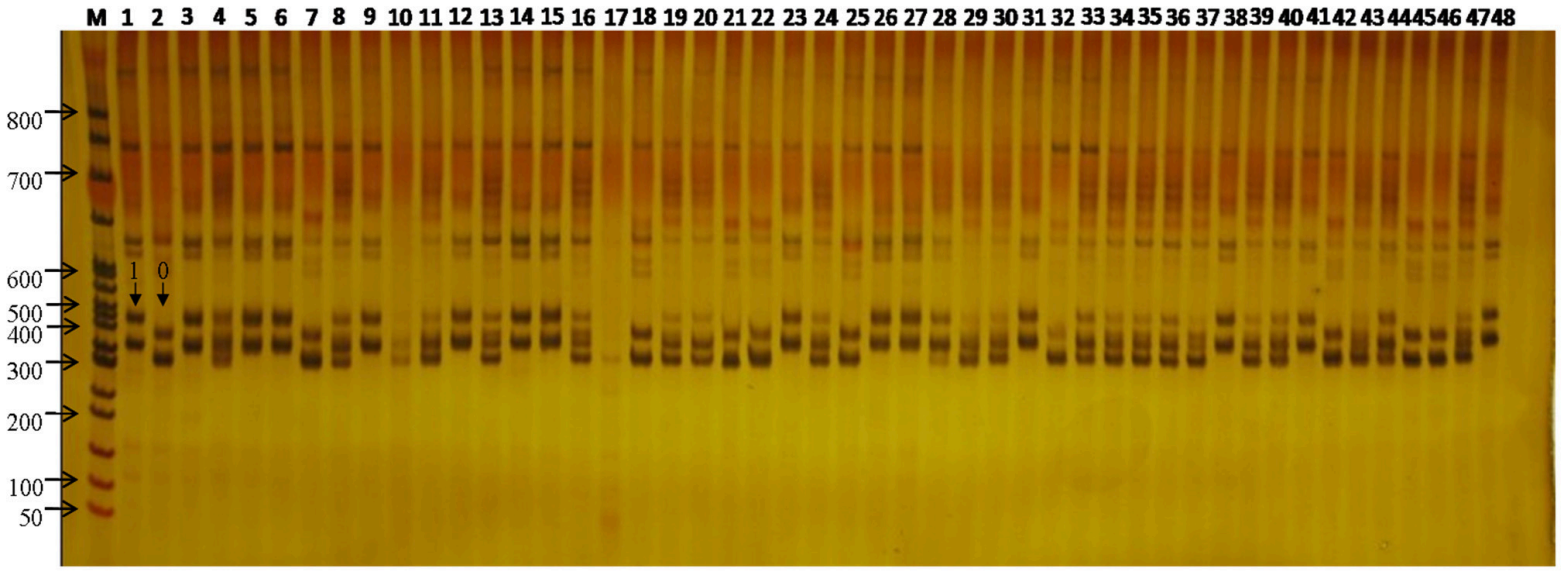
**SSR variation at the WSSR11 locus across the 48 flax**. M, DL3000 DNA ladder Marker; 1–48, each represents one of the 48 flax cultivars/accessions. The arrow showed SSR loci genotyped for 0 and 1 in the figure.

**Figure 3:**
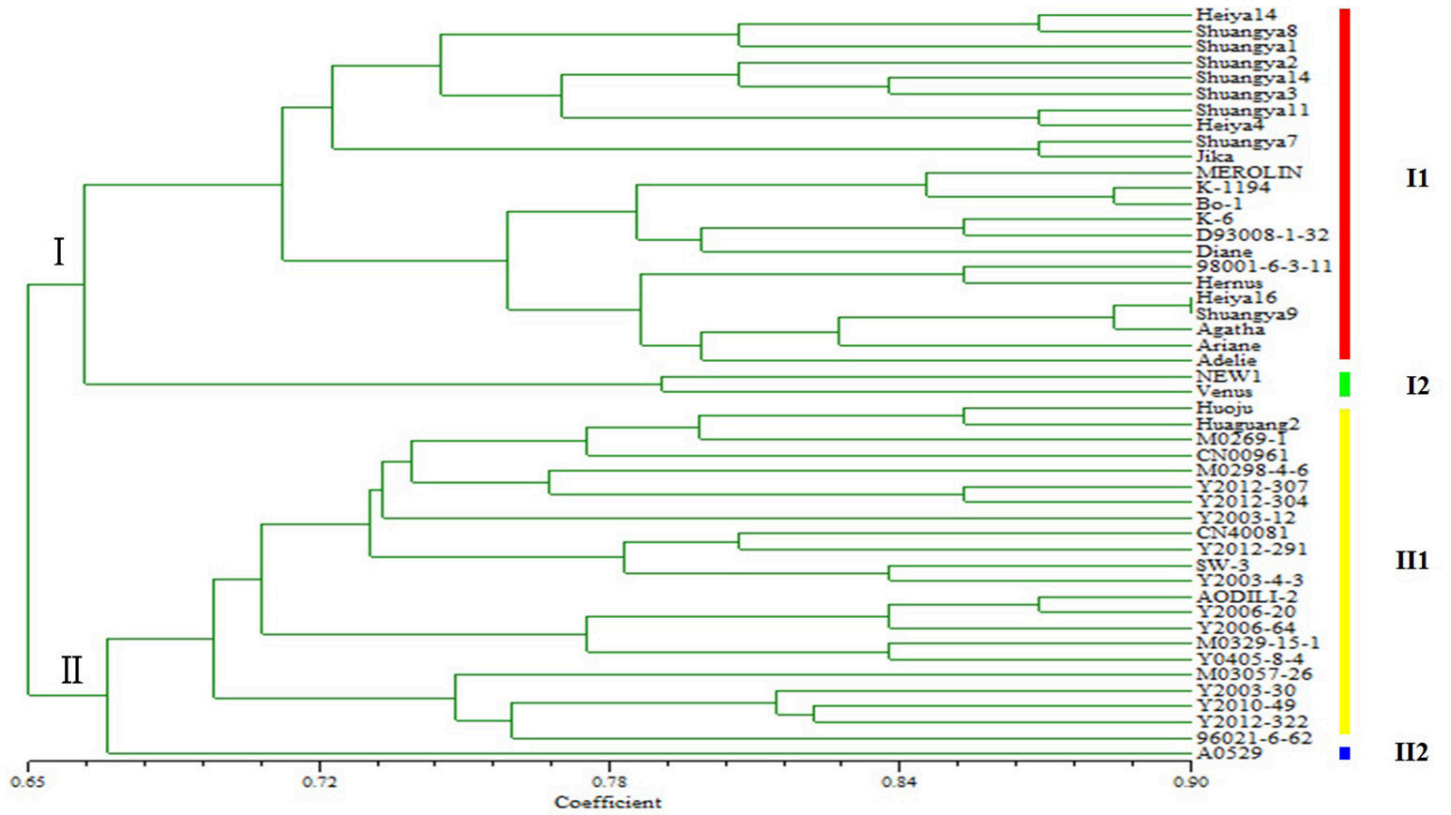
**Genetic diversity of 48 flax cultivars/accessions based on SSR markers**.

Genetic diversity is a result of gene evolution and is a necessary foundation for development of breeding programs to achieve desired genetic improvements of crops. In order to understand the genetic background of flax cultivars, 1006 polymorphic loci were detected among 48 flax varieties (Figure 2). Correlation of the polymorphic markers to the 48 flax varieties resulted in their classification into two groups; one group included 25 fiber cultivars and the other group included 23 linseed cultivars, in general agreement with the known dendrogram for fiber and linseed flax varieties (Table 1). Interestingly, all 25 fiber cultivars were clustered into the same group with the Shuangya series accessions cluster, while the 23 linseed cultivars clustered into a separate group. Also of note, it is clear that two cultivars “NEW1” and “Venus” exhibited genetic backgrounds distinct from the other fiber cultivars (Figure 3). Similarly, cultivar “A0529” exhibited a different genetic background from the other oilseed cultivars (Figure 3). Therefore, these three cultivars, “NEW1,” “Venus,” and “A0529,” have the greatest potential value for use in flax breeding programs.

The repeat characteristics of the SSRs discovered in this work should facilitate future SSR discovery. In agreement with our results, previous studies of SSRs of multiple varieties across multiple crop species have demonstrated that dinucleotide and trinucleotide repeat motifs are the major types of repeat motifs, although predominant motifs varied between species (Varshney et al., 2002; Kumpatla and Mukhopadhyay, 2005). Trinucleotide and dinucleotide motifs were the most abundant in fiber flax, as in Arabidopsis (Tian et al., 2004; Mun et al., 2006), soybean (Tian et al., 2004; Hisano et al., 2007), rice (Mun et al., 2006), pea (Gong et al., 2010), wheat (Yu et al., 2004; Peng and Lapitan, 2005), barley (Varshney et al., 2006), and citrus (Chen et al., 2006). Next in abundance were tetranucleotide, pentanucleotide, and hexanucleotide motifs, which together represented <10% of the SSRs identified. These results have been confirmed in other crops, including flax (Cloutier et al., 2009, 2012a). Therefore, these results showing frequency distributions of SSRs in crop genomes are mutually consistent.

This study also demonstrates that molecular markers, especially SSRs, are valuable because they can distinguish between different germplasm within a single species (Soto-Cerda et al., 2011b). In the present study, fiber and oil types of flax were distinguishable using SSR markers, as well as within these groups. In group I fiber flax varieties, although the variety “NEW1” was introduced from Holland and “Venus” was introduced from France, they clustered in the same subgroup, indicating that they share a similar genetic makeup (I2), but were more distantly related to the other varieties in subgroup one (I1), including the “Shuangya” and “Heiya” series cultivars. The “Shuangya” series cultivars are clustered tightly together, suggesting they share genetic similarity (with the exception of “Shuangya 9”). In contrast, the “Heiya” series cultivars are loosely clustered in the subgroup, suggesting a more diverse background. These results are in contrast to the results of a previous study (Li, 2011) that demonstrated that “Shuangya9” and “Heiya 16” clustered closely together, suggesting high genetic similarity. The disparity between these results might be explained by the fact that both cultivars were both adapted for optimal growth under similar environmental conditions of northeastern China; the results may reflect different marker biases between the two studies. Therefore, due to this discrepancy, in breeding programs we would use only one of these cultivars as donor germplasm.

Additional discrepancies need to be resolved regarding relative SSR motif frequencies for fiber vs. oilseed flax. Here, trinucleotide motifs were the most abundant motifs (56.10%), followed by the dinucleotide motifs (35.23%); tetranucleotide, pentanucleotide, and hexanucleotide motifs, which accounted for 8.67% of all SSRs identified. In addition, AT/TA dinucleotide repeats and CTT/GAA trinucleotide repeats were the predominant motifs observed in novel SSRs for fiber flax. These findings differ from former reports showing that AG/GA dinucleotide repeats and GAA/AAG trinucleotide repeats were predominant in EST-SSRs from linseed flax (Cloutier et al., 2009). The possible reason for this disparity may be due to the differences between the fiber flax SSRs studied here and oilseed flax SSRs studied in that work. Further research is needed to determine if SSR markers developed to distinguish between varieties of fiber flax may be transferable to linseed flax, as well as to other species of the same genus or closely related genera (Konishi et al., 2006; Tang et al., 2006). Of special note, in the oil flax group (II), the majority of cultivars clustered tightly together as one subgroup (II1). Only the Russian cultivar “A0529” occupied subgroup (II2). Due to its unique genetic makeup, this germplasm may hold particular value for use in genetic improvement of oil flax.

## Conclusion

We have developed 1574 novel SSRs in flax using reduced representation genome sequencing. We then used 62 of the selected sites to design primers for assessment of the genetic diversity among 48 flax varieties. The results indicated that the SSRs can be used to accurately separate flax varieties into two groups corresponding to the fiber and linseed categories. These new SSRs will play a critical role in genetic analysis, construction of linkage groups, quantitative trait loci mapping, and association mapping of flax and other crops.

## Author contributions

JW, QZ, GW, and SZ performed the experiments and bioinformatics analyses. JW wrote the paper. TJ directed the research. All authors read and approved the final manuscript.

### Conflict of interest statement

The authors declare that the research was conducted in the absence of any commercial or financial relationships that could be construed as a potential conflict of interest.

## Supplementary material

Supplementary Table 1 **All the SSRs developed in this study**. Accession number, SSR type, repeat motif, forward/reverse primer, and product size are represented.

Supplementary Table 2 **The redundant sequences to previous works**. Accession Numbers represented the redundancy sequence in recent study; Reported Accession represented the accession of reported in Nt database; And the identity of the redundancy sequence in recent study with the reported sequence in Nt database were listed.

Supplementary Table 3 **Summary of polymorphism SSR markers used in genetic diversity analysis**. The 62 Genomic SSR primers list including primer ID, repeat motif, forward/reverse primer sequences, annealing temperature, expected fragment size, gene diversity(H), PIC, Ne (1964), Shannon's information index(I).

Supplementary Table 4 **The polymorphism of the selected markers among the flax varieties**.
